# Complementary system vs conventional trifocal intraocular lens: comparison of optical quality metrics and unwanted light distribution

**DOI:** 10.1097/j.jcrs.0000000000001082

**Published:** 2022-12-17

**Authors:** Tadas Naujokaitis, Gerd U. Auffarth, Ramin Khoramnia, Grzegorz Łabuz

**Affiliations:** From The David J. Apple Center for Vision Research, Department of Ophthalmology, University of Heidelberg, Heidelberg, Germany.

## Abstract

To evaluate the ARTIS Symbiose complementary intraocular lens (IOL) system, consisting of the MID and PLUS models, in comparison with a conventional trifocal IOL (AcrySof IQ PanOptix).

**Setting:**

The David J. Apple Center for Vision Research, Department of Ophthalmology, University of Heidelberg, Heidelberg, Germany.

**Design:**

Laboratory investigation.

**Methods:**

Modulation and phase transfer functions were obtained in polychromatic light using an optical bench setup. Simulated visual acuity (VA) values were derived from optical quality metrics weighted by neural contrast sensitivity. United States Air Force (USAF) chart images were acquired and processed. Furthermore, the light distribution beyond the center of a polychromatic point spread function was assessed.

**Results:**

The peak simulated VA values of ARTIS Symbiose MID were at 0 diopters (D) of defocus (−0.02 logMAR) and at −1.5 D (0.00 logMAR); of ARTIS Symbiose PLUS, they were at 0 D of defocus (−0.01 logMAR) and at −2.5 D (0.01 logMAR). AcrySof IQ PanOptix demonstrated 3 peaks: at 0 D of defocus (−0.02 logMAR), at −1.75 D (0.03 logMAR), and at −2.5 D (0.02 logMAR). The summation of USAF chart images in the simulated binocular IOL system produced a slightly better image quality at −1.0 D and −1.5 D than AcrySof IQ PanOptix. The IOLs yielded comparable light spread across the studied range except for a localized intensity spike of the ARTIS Symbiose IOLs.

**Conclusions:**

The complementary IOL system may yield better monocular intermediate VA compared with the conventional trifocal IOL. However, the effect of binocular summation in terms of VA and the perception of photic phenomena still needs to be investigated.

## Abstract 2

The complementary IOL system may yield better monocular intermediate VA compared with the conventional trifocal IOL. However, the effect of binocular summation still needs to be investigated.

Presbyopia-correcting intraocular lenses (IOLs) have evolved from bifocal models first introduced in the late 1980s to today's more complex optical designs.^1,2^ The increasing importance of the intermediate range gave rise to trifocal technology.^3^ The latest developments in multifocal IOLs led to improved optimization of the light distribution and fewer photic phenomena.^4^

Monovision is an alternative approach for presbyopia correction, in which the dominant eye receives a monofocal IOL with the power for uncompromised distance vision while the nondominant eye is left myopic.^5,6^ The clinical studies of Goldberg et al. and Hayashi et al. provided evidence for a general acceptance of differences in image quality in monovision patients.^6,7^

In addition to multifocal IOLs and monovision, there is a mix-and-match approach, also known as blended vision, in which each eye receives a different IOL model, for example, 2 bifocal IOLs with different add powers or a bifocal and an extended-depth-of-focus (EDOF) IOL. Favorable outcomes of the mix-and-match procedure have been reported in various clinical studies.^2,8–10^ However, it should be noted that IOLs are primarily designed for bilateral implantation of the same model. As a result, there is no standardized mix-and-match method, which makes an interstudy data comparison challenging.

The approach of implanting 2 binocularly optimized IOLs, the ARTIS Symbiose MID (MID; Cristalens Industrie) and the ARTIS Symbiose PLUS (PLUS; Cristalens Industrie), may encourage a transition from a nonstandardized mix-and-match approach to a system of 2 IOLs that are specifically designed to complement each other.^11,12^

In this laboratory study, we compared the performance on the optical bench of the MID and PLUS IOLs with an established trifocal IOL, the AcrySof IQ PanOptix (PanOptix; Alcon Laboratories, Inc.), and simulated their binocular image quality.

## METHODS

### Intraocular Lenses

The following IOL models were investigated in this study: ARTIS Symbiose MID, ARTIS Symbiose PLUS, and AcrySof IQ PanOptix. All IOLs had the nominal refractive power of +20.0 diopters (D). Two samples per model were measured, and the results were averaged.

The ARTIS Symbiose MID and the ARTIS Symbiose PLUS IOL models are made from hydrophobic acrylic material. The anterior surface of the lens features a diffractive pattern. In addition to a distinct far focus, the MID features an EDOF profile for intermediate vision and the PLUS for near vision. The EDOF effect is achieved by combining more than one addition.^13^ The IOLs are designed to complement each other when implanted binocularly, with the resulting continuous zone of focus from the intermediate to near distance.^11^ An aspheric optical design has a negative spherical aberration (SA) of −0.23 μm to partially correct for a positive SA of the cornea.^11^ The refractive index of the lens is 1.54.^13^

The AcrySof IQ PanOptix is a hydrophobic acrylic IOL with a quadrifocal diffractive optic. The first 3 nonsequential diffraction orders distribute light rays to the far, intermediate, and near foci, with the 4th order reinforcing far vision. The aspheric design has a negative SA of −0.10 μm. The refractive index of the lens is 1.55.^14,15^

### Optical Metrology

The measurements were performed using the OptiSpheric IOL PRO 2 (Trioptics GmbH) device.

The optical quality assessment was performed in polychromatic light by using a spectral filter to simulate the spectral sensitivity of the human eye based on the findings of the Commission Internationale de l'Éclairage.^16^ The polychromatic light was chosen to simulate the clinical visual acuity (VA) testing conditions. The aperture size of 3 mm and 4.5 mm at the IOL plane was used. The model cornea had 0.28 μm of SA at 5.15 mm as described by the ISO standard.^17^ The longitudinal chromatic aberration of the model eye without the IOL was 1.04 D between 480 and 644 nm.^18^

The through-focus (TF) modulation transfer function (MTF) curves measured at the spatial frequency of 50 line pairs per millimeter (lp/mm) were used to determine the best focus at each of the IOL foci: the far, intermediate, and near foci of PanOptix, the far and intermediate foci of MID, and the far and near foci of PLUS. When MID is implanted in one eye and PLUS is implanted in the other eye, 3 foci emerge: far, intermediate, and near. To evaluate the optical quality of both IOLs at these 3 positions, we used the secondary focus of MID and PLUS as a reference point for intermediate and near range, respectively.

The optical quality measurements included the MTF and the phase transfer function (PTF) at the far, intermediate, and near foci at the aperture size of 3 mm and 4.5 mm at the IOL plane. The obtained sagittal and tangential MTF and PTF values were averaged. In addition, MTF and PTF were obtained within the defocus range of +0.5 to −3.5 D at the spectacle plane, where 0 D represents the distance focus of the IOL. The resolution of 0.25 D and the aperture size of 3 mm were used.

The weighted optical transfer function (wOTF) was calculated from the MTF and PTF values weighted by the neural contrast sensitivity function (CSF) as proposed by Alarcon et al. and used in our previous study:

$$\text{wOTF}=\frac{d}{150}\sum_{f=1}^{\frac{150}{d}}\text{MTF}\left(fd\right)\cos\left(\text{PTF}\left(fd\right)\right)\text{CSF}{\left(fd\right)}_{\text{neural}}$$

where *d* is the spatial frequency resolution of 1 lp/mm.^19–21^ We compared the change of the wOTF to the power of *b* = −0.36 with defocus among the studied IOL models because this parameter has shown a strong correlation with clinical VA.^19,21,22^ The value of *b* was derived from matching the wOTF metric with the postoperative visual outcomes in pseudophakic patients in a study by Alarcon et al.^19,22^

### United States Air Force Resolution Chart Images

For each IOL, the United States Air Force (USAF) resolution chart images were obtained within the same defocus range at 3 mm. To simulate vision with the ARTIS Symbiose binocular system, a quadratic summation of the MID and PLUS USAF resolution chart images was performed.^23^ The method is not suitable to simulate binocular vision in bilateral implantation of the same IOL model because it produces images identical to monocular ones. Because of this limitation, no binocular simulation is provided for the PanOptix IOL.

### Unwanted Visual Effects

We acquired images of a 0.1 mm pinhole through the IOLs to obtain a point spread function (PSF). The pinhole was illuminated from the back by the polychromatic light source, which is described above. This allowed a comparison of the light distribution from the IOLs beyond the PSF center at the 4.5 mm aperture.^21,24,25^ To extend the dynamic range of the 8-bit OptiSpheric IOL PRO 2's camera by 4 orders, multiple images were taken at different shutter times and combined. We compared the logarithmic normalized light intensity across the PSF cross-sections plotted over the angle in arcmin from the PSF center.

### Data Analysis

A custom-made software created in MATLAB (Mathworks, Inc.) was used for data analysis and image processing.

## RESULTS

### Optical Quality Assessment

**Figure 1.**
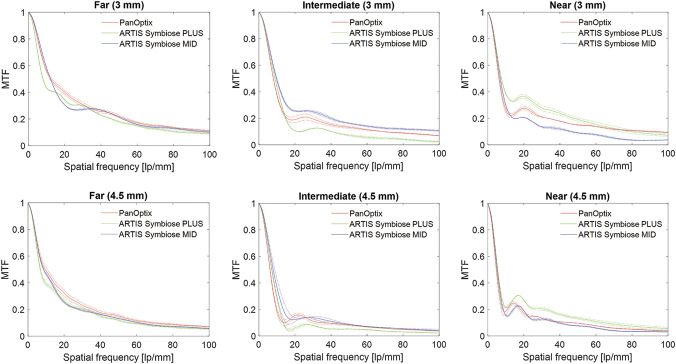
MTF levels at the far, intermediate, and near focus of the studied IOLs at 3 mm and 4.5 mm pupil size. Two samples per model were measured: the *dotted lines* show the values of each lens sample separately; the *solid lines* refer to the mean of 2 samples of the same IOL model. MTF = modulation transfer function

Figure 1 shows the study IOLs' MTF levels at far, intermediate, and near focus at 3.0 mm and 4.5 mm pupil sizes. At 3 mm aperture, at the far focus, MID and PanOptix demonstrated comparable MTF levels with a slightly better performance of the latter at around 20 lp/mm. The MTF of PLUS was minimally lower at nearly all spatial frequencies. At the intermediate focus, MID produced higher MTF values than the other studied IOLs, whereas PLUS outperformed the others at the near focus. At the pupil size of 4.5 mm, all IOLs had lower MTF values than at 3.0 mm, and the differences between the models were less pronounced.

**Figure 2.**
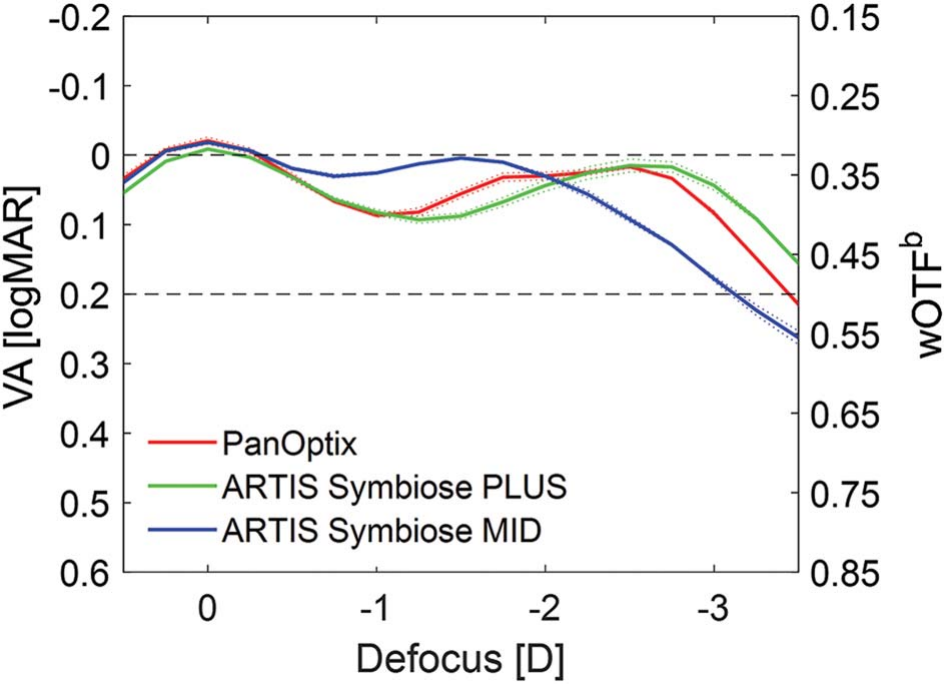
Simulated VA values and the corresponding values of the weighted optical transfer function to the power of *b* = −0.36 (wOTF^b^) at the defocus between +0.5 D and −3.5 D at the spectacle plane. Two samples per model were measured: the *dotted lines* show the values of each lens sample separately; the *solid lines* refer to the mean of 2 samples of the same IOL model. wOTF = weighted optical transfer function

The simulated VA defocus curves of the studied IOLs with the corresponding values of the wOTF to the power of *b* = −0.36 are shown in Figure 2.

The simulated VA peaks of MID and PanOptix IOLs were similar at zero defocus, whereas the peak of PLUS was slightly lower. In the range between 0 D and −1 D, the MID's curve minimally declined with a secondary peak at about −1.5 D. This lens outperformed the other 2 at approximately −0.5 to −2 D. At the defocus of −2 D, all the studied IOLs had nearly identical values. The simulated VA of both PLUS and PanOptix similarly showed a relatively sharp decline with the valley at about −1.25 D. Between −1.75 D and −2.75 D, the PanOptix's optical quality improved, showing an extended (from intermediate to near) focus area, followed by a sharp decline after passing −3 D. PLUS demonstrated its secondary peak at −2.5 D and a monotonous optical quality deterioration at higher defocus.

All the studied IOLs had the simulated VA of 0.2 logMAR or better throughout the range of +0.5 to −3 D and slightly negative simulated logMAR VA values (range −0.01 to −0.03 logMAR) at no defocus. The peak predicted VA values of MID were at no defocus (−0.02 logMAR) and at −1.5 D (0.00 logMAR), of PLUS at no defocus (−0.01 logMAR) and at −2.5 D (0.01 logMAR), and of PanOptix at no defocus (−0.02 logMAR) and at −2.5 D (0.02 logMAR).

### USAF Resolution Chart Images

**Figure 3.**
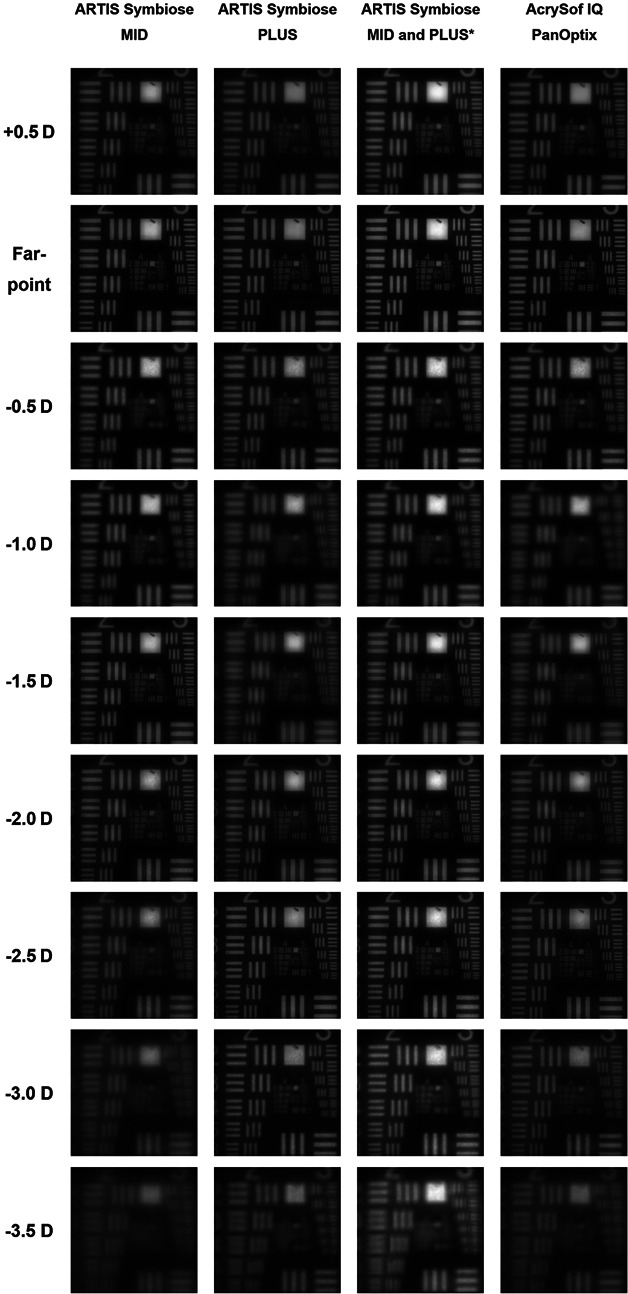
USAF resolution chart images recorded through the MID, PLUS, and PanOptix IOLs at a defocus range of +0.5 to −3.5 D and the 3 mm aperture. *To simulate vision with the ARTIS Symbiose binocular system, a quadratic summation of the MID and PLUS USAF resolution chart images was performed.

Figure 3 shows USAF resolution chart images recorded at a defocus range of +0.5 to −3.5 D in 0.5 D steps. MID had the best image quality at 0 D and approximately −1.5 to −2.0 D, PLUS at 0 D and approximately −2.5 D, and PanOptix at 0 D and approximately −2.5 D. The quadratic summation of the images of MID and PLUS is also presented. These images are generally comparable with those of PanOptix, except for −1.0 D and −1.5 D, where the summation produced slightly sharper images (higher spatial frequency).

### PSF Analysis

**Figure 4.**
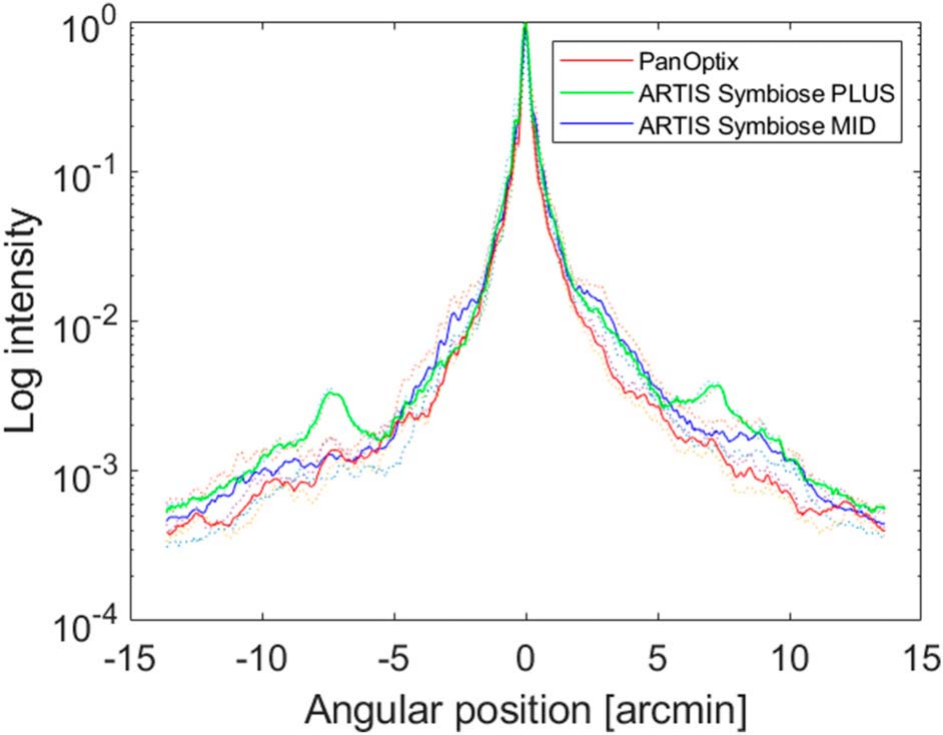
Cross-section of the point spread function at the pupil diameter of 4.5 mm. Two samples per model were measured: the *dotted lines* show the values of each lens sample separately; the *solid lines* refer to the mean of 2 samples of the same IOL model.

The cross-sections of the polychromatic PSF at the pupil size of 4.5 mm indicate slightly higher intensity of the PSF approximately between 2 and 5 arcmin from the center for the MID IOL, compared with the other 2 IOL models. At approximately 5 arcmin from the center, all the IOLs show a similar intensity of the PSF. Between 5 and 10 arcmin from the center, the PLUS IOL shows the highest intensity of the PSF. At more than 10 arcmin from the center, the PSF intensity decreases to low levels, and the differences between the IOL models become less pronounced (Figure 4).

## DISCUSSION

Multifocal IOLs have become an established method to achieve spectacle independence after cataract surgery. Although currently available multifocal IOLs provide a high score of patient satisfaction, they have several limitations.^2^ Decreased VA between designed foci and the occurrence of photic phenomena such as halos may still cause dissatisfaction.^2,4^ In our laboratory study, we investigated how these issues affect the ARTIS Symbiose IOL system compared with an established PanOptix trifocal IOL. Using the optical bench measurements and computational methods, VA was simulated across a wide range of defocus. In addition, the unwanted light distribution at an increased pupil size was assessed.

Zapata-Díaz et al. compared PLUS and MID with, among others, PanOptix in a laboratory study.^11^ Although PLUS and MID separately had a lower total depth of focus (range in which the TF MTF at 50 lp/mm is 0.15 or greater) than PanOptix, bilateral implantation of the ARTIS Symbiose IOLs could theoretically provide the total depth of 2.90 D, higher than the one of PanOptix (1.90 D).^11^ Zapata-Díaz et al. investigated their IOLs in monochromatic light using a single frequency. By contrast, we analyzed the IOLs in polychromatic light, which may influence the depth of focus and used the wOTF to simulate the clinical performance of the IOLs.^26^ Given the multifrequency character of the wOTF metric, the visual quality decrease of the PanOptix was less pronounced than presented by Zapata-Díaz et al. with their single-frequency MTF approach. Our result showed an intermediate VA level that is not worse than 0.1 logMAR, which agrees with clinical study results, indicating a focus extension of the PanOptix IOL that spans over a comparable defocus range as the complementary system.^27^ The latter, however, offered slightly extended near distance because of the secondary peak location of the PLUS IOL in the binocular simulation.

The ARTIS Symbiose is a new IOL system, and the clinical data describing the performance of these IOLs are scarce. In the clinical study by Zapata-Díaz et al. twenty patients had MID in the dominant eye and PLUS in the fellow eye.^12^ Monocular defocus curves obtained 1 to 2 months postoperatively were, in many aspects, comparable with those derived from our TF optical measurements. In that clinical study, the MID showed a secondary VA peak at −1.75 D. For the PLUS, it was at −2.25 D. Both defocus curves intersected at −2 D, which agrees with our laboratory findings. The clinical VA values at the peaks were close to the simulated VA values. However, our simulated defocus curves seem to overestimate the VA values between the peaks, resulting in a flatter profile compared with the clinical one. We consider that these observations point out the need to refine the model for predicting VA with the ARTIS Symbiose IOLs. The overestimation of VA values in the model could be partly explained by the fact that the simulated values were compared with monocular defocus curves, whereas the current model has been demonstrated to correlate with binocular VA.^19^ In the ARTIS Symbiose IOL system, different IOL models are implanted in each eye, and therefore, binocular VA for a specific IOL model cannot be tested.

The binocular defocus curve reported by Zapata-Díaz et al. showed a marked VA improvement compared with the monocular ones.^12^ Of interest, a positive effect of the binocular summation was observed not only when monocular VA values were similar but also when there was a wider gap between the 2 models. As an example, at −1.25 D, the MID's VA was 0.04 logMAR and 0.17 logMAR for the PLUS model. This improved binocularly to −0.02 logMAR, which for the MID model slightly exceeded the 7% binocular improvement expected in healthy eyes.^28^ Further research is needed to determine the binocular summation coefficient for the ARTIS Symbiose IOLs.

In a previous laboratory study, we evaluated the light distribution using the ray propagation imaging as well as monochromatic (green light) optical performance of the PanOptix and found 3 distinct foci: the highest amount of the light energy was distributed to the far focus (MTF value of 0.371 at 3 mm and a spatial frequency of 50 lp/mm), a lower amount of the light energy to the near focus (MTF value of 0.172), and the least amount of the light energy to the intermediate focus (MTF value of 0.164).^14^ Lee et al. reported similar findings after their assessment of PanOptix.^29^ In this study, however, we observed lower values, which resulted from the impact of longitudinal chromatic aberration on polychromatic image quality and a high dispersion level of AcrySof material.^18^

Given that PanOptix was launched to the market earlier than the ARTIS Symbiose IOLs, there have been more studies published that analyzed the postoperative outcomes in patients with PanOptix.^9,10,27,30–32^ In 1 multicenter clinical trial, the binocular defocus curves of both PanOptix and AT LISA tri 839MP (Carl Zeiss Meditec AG) ranged from 0.1 to 0.0 logMAR between 0 D and −3 D; however, PanOptix demonstrated better VA results between −1.5 D and −2.5 D.^27^ The PanOptix defocus curves were similar in other clinical studies.^31,32^ Kohnen et al. published both monocular and binocular clinical defocus curves of PanOptix. The improvement due to binocular summation ranged from 0.01 to 0.06 logMAR.^27^ He et al. simulated PanOptix's defocus curve using the computer software and found that it was generally consistent with the clinical defocus curve.^33^ In principle, discrepancies between the laboratory-based simulations and clinical results could arise due to the fact that the laboratory measurements are performed under standardized conditions, lacking the intersubject variability present in clinical data. However, our simulated defocus curves were generally within the expected clinical range, and any discrepancies such as the difference between VA at no defocus and at −1.0 D of defocus (simulated 0.11 logMAR vs clinical 0.14 logMAR) could be explained by the variability observed in the normal population.^33^

The clinical performance of the bilateral implantation of PanOptix has also been compared with TECNIS Symfony ZXR00 (Johnson & Johnson Vision), an EDOF IOL, in the dominant eye and TECNIS ZMB00/ZLB00 (Johnson & Johnson Vision), a bifocal IOL with the addition of +3.25 D, implanted in the nondominant eye.^9,10^ The combination of ZXR00 and ZMB00/ZLB00 IOLs is similar to the ARTIS Symbiose IOL system: in addition to the far focus, one IOL is optimized for the intermediate distance and the other one for the near distance. The binocular defocus curves reported by de Medeiros et al. revealed a (statistically) better performance of the mixed implantation of ZXR00 and ZMB00 IOLs than the bilateral implantation of PanOptix throughout the range of defocus from 0 to −3.5 D, except for −2.0 D, where PanOptix had a superior VA, and −2.5 D, where the difference between the groups was not statistically significant.^9^ The study by Song et al. found the binocular defocus curve to only differ between −0.50 D and −1.00 D, where the mix-and-match group showed superior results, and at −4.0 D, where the PanOptix group was superior to the mix-and-match group.^10^

Photic phenomena such as halo and glare may not be well tolerated by every pseudophakic patient, which, in some cases, may necessitate IOL explantation.^34^ The design of the EDOF and multifocal IOLs may lead to increased photic phenomena perception. Glare occurs due to light scattering by imperfections in the optical media, one of which may be the edges of the diffractive rings in diffractive multifocal IOLs.^35^ Halo is another type of photic phenomenon caused by defocused light. In EDOF and multifocal IOLs, a part of the light is defocused, which leads to increased halos. The greater the defocus (ie, addition in the multifocal IOLs), the higher the diameter and the lower the intensity of the halo.^2^ This was generally consistent with our halo simulation using the PSF. The results of our PSF measurements suggest that the PLUS IOL may have a larger but less intense halo than the MID IOL. However, there is significant variability in photic phenomena perception among individuals, which may be due to the differences in neuronal processing.^2^ For this reason, caution is necessary when applying the laboratory findings on photic phenomena to the clinical setting.

A limitation of our study is that no statistical analysis could be performed because of the low sample size. However, the inclusion of 2 IOLs per model was motivated by high manufacturing standards of contemporary diffractive IOLs with a proven reproducibility of the optical quality parameters.^36^ Furthermore, the laboratory's ISO standards–compliant industrial optical bench also ensures rigorous measurement repeatability, which has been shown in an earlier study.^18^ The same conclusion can be drawn from the current results because the quality metrics of 2 samples (each marked as dotted lines in Figures 1, 2, and 4) indicate a low intersample variability. Still, the comparison of the IOLs with different nominal powers using statistical methods is of interest and requires further research.

The wOTF-based VA simulations demonstrate good agreement with clinical results found in the literature. The simulated VA analysis indicates that patients implanted with MID in one eye, and PLUS in the contralateral eye, may have superior monocular intermediate VA in the eye implanted with MID than those with PanOptix. Higher tolerance to defocus of PLUS may translate into better monocular VA at higher defocus but may also result in slightly higher halo intensity observed monocularly. Clinical studies are necessary to confirm these observations. Likewise, more research is needed to better understand the effect of binocular summation in both ARTIS Symbiose and PanOptix IOLs in terms of VA and the perception of photic phenomena.

**WHAT WAS KNOWN**

- A nonstandardized mix-and-match procedure, in which different IOL models are implanted, has been shown to provide good bilateral VA outcomes offering a high acceptance level.
- A new blended vision system consists of 2 IOLs that are specifically designed to complement each other and create binocular trifocality.

**WHAT THIS PAPER ADDS**

- VA derived from weighted optical transfer function demonstrates a good agreement with clinical results found in the literature and its usefulness to predict postoperative defocus curves of trifocal IOLs.
- The VA simulations based on optical quality measurements indicate better monocular intermediate VA in the complementary system than the conventional trifocal IOL.
